# Vascular Inflammatory Markers as Predictors of Peripheral Arterial Disease Patients’ Quality-of-Life Changes after Endovascular Treatment

**DOI:** 10.3390/jcm12103412

**Published:** 2023-05-11

**Authors:** Agnieszka Wachsmann-Maga, Mikołaj Maga, Romuald Polczyk, Aleksandra Włodarczyk, Patrycja Pasieka, Karol Terlecki, Paweł Maga

**Affiliations:** 1 Department of Angiology, Faculty of Medicine, Jagiellonian University Medical College, 31-008 Kraków, Poland; 2 Clinical Department of Angiology, University Hospital in Kraków, 30-688 Kraków, Poland; 3 Department of Rehabilitation in Internal Diseases, Faculty of Health Sciences, Jagiellonian University Medical College, 31-008 Kraków, Poland; 4 Institute of Psychology, Jagiellonian University, 30-060 Kraków, Poland; 5 Department of Dermatology, Jagiellonian University Medical College, 31-008 Kraków, Poland; 6 Department of Vascular Surgery and Angiology, Medical University of Lublin, 20-081 Lublin, Poland

**Keywords:** peripheral arterial disease, quality of life, inflammation, endothelium, eicosanoids, leukotrienes, thromboxanes

## Abstract

The association between chronic inflammation and depression, anxiety, anhedonia, and quality of life (QoL) has been recently emphasized. However, the pathophysiology of this relationship remains unsolved. This study aims to assess the dependence between vascular inflammation represented by eicosanoid concentration and quality of life in patients with peripheral arterial disease (PAD). A total of 175 patients undergoing endovascular treatment due to lower limbs ischemia were covered with eight years of observation after the endovascular procedure, including ankle-brachial index (ABI), color Doppler ultrasound examination, urinary leukotriene E4 (LTE4), thromboxane B2 (TXB2) and 5-Hydroxyeicosatetraenoic acid (5-HETE) measurement and quality-of-life assessment with VascuQol-6. The baseline concentrations of LTE4 and TXB2 reversely correlated with preoperative VascuQol-6 and were predictive of the postoperative values of VascuQol-6 at each follow-up. At every follow-up timepoint, the results of VascuQol-6 reflected the LTE4 and TXB2 concentrations. Higher concentrations of LTE4 and TXB2 were correlated with lower life quality during the next follow-up meeting. Changes in VascuQol-6 at eight years vs. preoperative values were reversely related to the preoperative concentrations of LTE4 and TXB2. This is the first study to confirm that changes in life quality in PAD patients undergoing endovascular treatment are highly dependent on eicosanoid-based vascular inflammation.

## 1. Introduction

Peripheral arterial disease (PAD) is an atherosclerosis-based civilization disease that affects more than 200 million people worldwide and has become an important issue of the healthcare system [1,2]. The first symptoms of PAD are most commonly lower limb cramps and intermittent claudication, escalating into resting pain localized in the lower extremities, then into unhealing wounds and lower limb necrosis [3]. Those ailments impair the ability to walk and conduct daily routines, significantly impacting the life quality of patients. There is strong evidence of the association between PAD and mood disorders [4,5]. According to the literature, patients with PAD quite frequently suffer from depression, anxiety, and anhedonia. The prevalence of psychiatric conditions is estimated to range from 11% to as high as 48% [6]. In addition, rates of aforementioned mood disorders are thought to increase with the severity of reported symptoms of PAD [5].

Recently, an association between chronic inflammation and quality of life has been afresh emphasized. However, the pathophysiology of this relationship remains unsolved. It has been shown that in patients with major depressive disorders, an increased level of cytokines such as IL-6, IL-1β, TNF alpha, and CRP is observed [7]. Similar results were obtained in the study considering patients with chronic low back pain, where an increased IL-6 level was observed among patients with higher pain severity and sleep problems in addition to a depressive mood [8]. Lowering the level of pro-inflammatory eicosanoids in patients with asthma also leads to quality-of-life improvement [9].

Even though the combination of dyslipidemia and inflammation is known to be the pathophysiological mechanism leading to atherosclerosis, all of the exact pathways and dependences are still not fully discovered and proven. In the last decade, the role of the inflammatory component was growing the attention of scientists [10,11]. New ways of inhibiting vascular inflammation were presented, showing promising clinical results, especially in improving outcomes in patients with coronary arterial disease [12,13]. Unfortunately, not all of the new pharmacotherapy pathways were equally effective in PAD patients [14]. The group of vascular inflammatory biomarkers that were hypothesized to play a significant role in the development of PAD are products of arachidonic acid, especially leukotrienes (LT) [15,16]. According to Maga et al., they are not only related to the occurrence of re-stenosis in PAD patients, but also the elevated LT values predict the incoming poor outcome of endovascular treatment [17]. The latest studies prove that increased concentrations of LT E4 and B4 directly impair the functions of endothelium by decreasing flow-mediated dilatation and increasing the stiffness of arteries [18].

This study aimed to examine the dependence between eicosanoids represented by leukotrienes E4 (LTE4), 5-Hydroxyeicosatetraenoic acid (5HETE), and thromboxane B2 (TXB2) corresponding to vascular wall inflammation and the dynamics of changes in life quality of patients undergoing endovascular treatment due to peripheral arterial disease.

## 2. Materials and Methods

### 2.1. Participants

Consecutive patients of both genders aged 45–85 years old undergoing plain balloon angioplasty or angioplasty combined with stenting due to peripheral arterial disease were enrolled in this prospective observational study. In accordance with inclusion criteria, all of the participants had chronic lower limb ischemia in stages 3–4 of the Rutherford classification. The ulcerations, acute lower limb ischemia, unstable coronary artery disease, signs of inflammation (such as skin infections, fever, or active wounds), and previously diagnosed asthma or currently ongoing neoplasm were excluded from participation in the study. Participants in which the drug-coated balloons were used or drug-eluting stents placed within the last 6 months were not included in the study.

**Figure 1:**
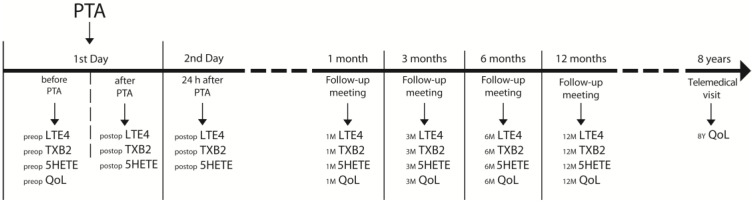
Study timeline.

All of the participants were covered with follow-up meetings in angiology outpatient 1, 3, 6, and 12 months after the endovascular procedure as well as a telemedical visit 8 years later, as presented in Figure 1.

### 2.2. Follow-Up Meetings Outcomes Assessment

Every follow-up meeting consisted of an interview with the participant, basic physical examination including a 4-point pulse assessment at lower limbs, and ABI measurement (Hadeco Doppler Bidop ES 100 V3 Bi-directional flowmeter, Kawasaki, Japan). Color Doppler ultrasound examination of lower limbs arteries was performed with two ultrasound units: Siemens Acuson Sequoia 512 with linear 15L8W and convex 6C2 transducers (Siemens Healthcare, Erlangen, Germany) and Hitachi Arietta 850 with linear L441 and convex C252 transducers (Hitachi, Tokyo, Japan). During a telemedical follow-up conducted 8 years after hospitalization, every participant was questioned with the prepared vascular interview, and the medical documentation on any hospitalizations was obtained from patients (Supplementary Figure S1).

### 2.3. Laboratory Measurements

Urine samples were taken from participants in the morning during hospitalization (before and after the endovascular procedure) and at 1, 3, 6, and 12-month follow-up meetings. They were centrifuged (10 min, 5000× *g*) and frozen at −70 °C. Leukotriene E4 (LTE4), thromboxane B2 (TXB2), and 5-Hydroxyeicosatetraenoic acid (5-HETE) urine concentrations were assessed in all patients using high-performance liquid chromatography-mass spectrometry recalculated by urine creatinine level. The detailed method of laboratory measurements was described in our previous articles [17,19].

### 2.4. Quality-of-Life Assessment

Participants’ quality of life was assessed by a disease-specific Vascular Quality-of-Life (VascuQoL-6) questionnaire, the shortened validated version of VascuQol-25 [20,21]. It assesses the life quality in 5 domains (activity, symptoms, activity, emotional, social, and pain) and also includes the summarized SCORE. The activity domain is based on 2 questions (numbers 1 and 3). Participants filled out the questionnaire on their own at hospitalization as well as during follow-up meetings. At telemedical visits, the questionnaire was distributed to patients and filled out via telephone conversation.

### 2.5. Statistical Analysis

Descriptive results were expressed as means and standard deviations (standard errors on the figures) and numbers and percentages. The assumption of normality was checked for all variables by means of visual inspections of histograms of distributions as well as the Shapiro-Wilk tests. Most variables showed a significant and considerable departure from normality. Most vascular inflammatory markers had a right-skewed distribution. We tried to correct that by means of a log transformation. The variables relating to the quality of life showed left-skewed distributions. The variables were reversed, log-transformed, and reversed back. In most cases, the skewness of the variables was reduced, although not eliminated at all. Indicators of the magnitude of change were computed by subtracting the results before the intervention from each respective time point.

In order to analyze the differences among time points, a repeated-measures ANOVA was applied, with multiple comparisons among time points adjusted by means of the Sidak method. As the assumption of sphericity was not met, Greenhouse-Geisser correction was applied. Pearson r correlations were used to analyze the relationships between the vascular inflammatory markers and quality of life.

### 2.6. Ethical Aspects

All of the enrolled patients have given informed consent to participate in this study. All participants were treated with the standard treatment procedures dedicated to patients with chronic lower limb ischemia, which was not modified due to participation in the study. The proper approval of the Jagiellonian University Medical College Bioethical Committee was obtained prior to the beginning of the study (1072.6120.365.2020), which was conducted in accordance with The Code of Ethics of the World Medical Association (Declaration of Helsinki) for experiments involving humans.

## 3. Results

### 3.1. Patients Characteristics

A total of 175 patients, mean age of 64.8 years old, were enrolled in the study, with a majority of males (68.6%). Most of the participants were in stage 3 in the Rutherford classification, and all of them underwent plain balloon angioplasty. The studied population was with a high burden of cardiovascular comorbidities, with 40% of them with coronary artery disease, 34% with diabetes type II, and 73% with hypertension. Detailed population characteristics are presented in Table 1. The primary patency of treated arteries was 79% (139) within 12 months. In 49 (28%) participants, reintervention due to hemodynamically significant arteries stenosis or occlusion was necessary. There were no major or minor amputations needed. Six patients died, and one (0.57%) was lost-to-follow-up. In the 8-year observation, 49 (28%) deaths were recorded, and an additional 19 participants (11%) were lost to observation.

### 3.2. Quality of Life and Its Changes after Endovascular Procedures

The mean summarized VascuQol-6 score elevated from 2.24 to 3.60 (*p* < 0.001). Those values did not change significantly during the 12-month observation, finally decreasing significantly to 2.92 after eight years (*p* < 0.001) but remained superior compared to the baseline level (*p* = 0.038) (Figure 1). This pattern of results was generally the same for every domain of life quality; in particular, one month after the endovascular procedures, the participants had a significant increase in every domain of life quality: activity (2.28 to 3.52), symptoms (2.31 vs. 3.43), emotional (1.78 vs. 3.64), social (2.34 vs. 3.70), and pain (2.43 vs. 3.78); all *p* < 0.001).

The general dynamics of pro-inflammatory biomarkers perioperative levels have been described previously, and their exact values are presented in Supplementary Table S1 and Figure S2 [17,19].

### 3.3. Preoperative Inflammatory Biomarkers and Life Quality

The baseline concentrations of LTE4 and TXB2 (respectively 149.82 ± 91.64 µg/mL and 1760.09 ± 2007.76 µg/mL) negatively correlated with the preoperative result of summarized preoperative VascuQol-6 (*p* < 0.01). The same phenomenon was not present in 5-HETE concentrations. Baseline concentrations LTE4 and TXB2 were predictive for the postoperative values of VascuQol-6 at each of the follow-ups for 12 months (both *p* < 0.001). There was no significant dependence between preoperative values of LTE4, TXB2, or 5HETE and eight-year observation life quality (*p* > 0.05). Preoperative 5HETE concentrations did not show prediction for VascuQol-6 results at any follow-up time points (Table 2).

### 3.4. Inflammatory Biomarkers and Life Quality during Follow-Up

At every single time point of the 12-month observation, the results of VascuQol-6 reflected the LTE4 and TXB2 concentrations. The same observation was not present for the 5HETE. Both LTE4 and TXB2 concentrations presented significant predictions for life quality in forthcoming months. The higher concentrations of LTE4 and TXB2 were correlated with lower life quality during the next follow-up meeting (Table 2).

LTE4 at each time point of postoperative observation also was related to the dynamics of changes in VascuQol-6 results (*p* < 0.001) (Table 3). It also presented predictive features at one month for three months of quality-of-life change (*p* < 0.001) and at three months for six months QoL (*p* < 0.01), when such dependence was not observed in case of 6- and 12-months observation (*p* < 0.05).

TXB2 concentrations were not related to the changes in VascuQol-6 at any time points of postoperative observation (*p* > 0.05), but its preoperative value was predictive for 1-month postoperative change in life quality (*p* < 0.01) (Table 3).

5HETE did not present any significant relationship with either the values or changes in quality of life (*p* > 0.05) at any observation time points (Table 2 and Table 3).

Values of VascuQol-6 at the eight years time point did not present any relationship with the LTE4, TXB2, or 5HETE concentrations (Table 2), but its eight-year vs. preoperative change was reversely related to the preoperative concentrations of LTE4 and TXB2 (*p* < 0.01) (Table 3). 

### 3.5. Impact of Changes in Inflammatory Biomarkers Concentrations on Quality-of-Life Changes during Follow-Up

The changes in concentrations of LTE4 and TXB2 compared to the preoperative values were accompanied by the reverse changes in quality of life at the same time points. (LTE4 1,3,6,12 M and TXB2 1,3 M *p* < 0.001; TXB2 6,12 M *p* < 0.01).

The changes in concentrations of LTE4 at one and three months of observations were predictive of reverse changes in quality of life in the following months *p* < 0.001). The same phenomenon was observed for TXB2 changes after one month *p* < 0.001). There was no significant correlation between changes in 5HETE concentrations and changes in quality of life. The changes of 5HETE did not present the predictive values for changes in life quality at the following them timepoints (Table 4).

## 4. Discussion

Analyses of life quality indicated that it rose considerably after the endovascular treatment, remained at this higher level for 12 months, and dropped during the following eight years but remained higher than before angioplasty. This applied to the general results on the VascuQol-6 questionnaire as well as its domains: activity (including the ability to walk), experienced symptoms, emotional concerns, participation in social activities, and pain. The general results on the VascuQol-6 questionnaire were expressed as means from the six questions, with the possible range from ‘1’, referring to the most severe problem (e.g., ‘All of the time’, ‘Couldn’t walk at all’), to ‘4’—no problem at all, e.g., ‘Not at all limited’, ‘No discomfort or stress’. Before the endovascular treatment, the mean on the VascuQol-6 was 2.24, that is, close to severe problems. After one month, the mean was 3.60, that is, somewhat between least severe problems and no problems at all. After eight years, the mean dropped somewhat but still was close to the least severe problems (2.92). This confirms that the endovascular treatment had a long-lasting positive impact on the well-being of the patients and is, along with previous reports [22,23].

The pattern of results of correlational analyses concerning the relationships between the inflammatory biomarkers and life quality was quite clear-cut. As for the LTE4 and TXB2, they were consistently related to life quality: at the preoperative level, at subsequent time points, and as changes from the baseline: the lower were LTE4 and TXB2, or the greater their decrease, the higher the quality of life. This did not concern the measurement performed eight years after endovascular treatment, probably because such a period was connected with a great number of possible influences on life quality after such a long time. Nether the less, the initial level of vascular inflammation represented by concentrations of TXB2 and LTE4 kept its predictive value for changes in life quality even over such a long time. This proves that vascular inflammation has a great impact on postoperative long-term outcomes, while so far, only predictions of short-time outcomes have been described [17,19]. Our analysis of the dependencies between eicosanoids and life quality was complex as we took under consideration not only the absolute values but also concentration changes and their dynamics. This is due to a lack of standardized cut-off points or normal basal levels of examined substances [24].

The exact mechanism regulating the impact of inflammation on the process of atherosclerosis remains the subject of discussion [16]. It is associated with endothelial dysfunction, but the role of interleukins and their inhibition has been emphasized again by the CANTOS study [13]. Even though the rate of MACEs was reduced by canakinumab, proving the importance of interleukins in coronary artery disease, the occurrence of peripheral arterial disease adverse events was not significantly minimalized [14]. This leads once again to the conclusion that the mechanism of PAD and CAD differ significantly, and further researches on the pathophysiology of those manifestations of atherosclerosis are essential in the development of future efficient treatment methods [11].

The eicosanoids have been hypothesized to be involved in the development of atherosclerosis plaque and to significantly impair the arteries functioning [25]. The direct product of arachidonic acid metabolism by 5-lipooxygenase, which is expressed in leukocytes and many other cells responsible for immune defense, is 5HETE, which is not a stable substance. It is easily metabolized into the leukotriene A4 starting the leukotrienes production pathway [26]. We hypothesize that its short time of duration is the biggest reason for the lack of any significant relations with quality-of-life outcomes. The LTs family includes five compounds: unstable LTA4 and LTB4, as well as cysteinyl leukotrienes, including LTC4, LTD4, and LTE4 [26]. The last one has become a useful biomarker of systemic LTs synthesis [27]. These pro-inflammatory agents take part in chemotaxis and are able to maintain inflammation [28]. This hypothesis was clinically supported by the Swedish report on the decreased vulnerability to CAD of asthmatic patients taking anti-leukotriene drugs [29]. A recent systematic review on the leukotrienes in atherosclerosis-based arterial diseases also proves that elevated LT concentrations are related to an increased risk of stroke, myocardial infarction, or the development of PAD [15]. In our previous studies, we presented that elevation of both LT E4 and LT B4 is related to the occurrence of re-stenosis and re-occlusions in patients after percutaneous transluminal angioplasty due to PAD [17,18].

There is evidence of the relationship between chronic inflammation and changes in life quality [30]. According to Nowakowski et al., patients suffering from chronic inflammation with elevated CRP concentrations have lower odds of reporting high QoL, especially in relational and emotional domains [31]. The impact of inflammation on the life quality decrease is observed in inflammatory-related diseases like rheumatoid arthritis [32], asthma [33,34], Crohn’s disease [35], ulcerative colitis [36], inflammatory bowel disease [37], and vulvodynia [38].

The dependence between elevated interleukins IL-6 and IL-1 and depressive disorders has been described [7]. As both of those cytokines play a significant role in atherosclerosis [39], an assumption that a higher rate of depression in patients with peripheral arterial disease occurs may also be related to the inflammatory background. Even though the inflammatory component of atherosclerosis has been known for a long time, there are no studies examining the direct impact of increased vascular wall inflammation on the life quality changes in patients with peripheral arterial disease. There are also studies proving that permanent stress enhances inflammation itself [40]. As a consequence, patients with a lowered quality of life due to their stress-causing disease are more vulnerable to a basal elevation of inflammation, which may further decrease their life quality [31,41]. Most of the studies analyze the general inflammatory biomarker—the CRP protein [42,43,44]. What is more, there are no studies at all examining the impact of inflammation on life quality in PAD patients. In our study, we focused on more peripheral arterial disease-specific inflammatory substances, which previously proved to have a direct influence on treatment outcomes [15]. In this study, we tried to limit factors influencing inflammation and eicosanoid concentrations and bias the results. Sirolimus, which is one of the substances used in drug-eluting stents (DES) and drug-coated balloons (DCB), has strong anti-inflammatory properties [45]. The use of drug-coated devices also requires prolonged dual antiplatelet therapy influencing the endovascular treatment outcomes significantly but also reducing vascular inflammation [46,47]. That is why we decided not to include patients undergoing endovascular treatment with the use of DEB or DCB.

The study has its limitations. Even though only four patients were previously diagnosed with depression, the history of antidepressant drug use of all participants is unknown, which could impact the obtained results. Only during the first 12 months patients were attending on-site follow-up meetings. After this time, observation was kept only by telephone contacts. In the meantime, multiple of them suffered from re-occurrence of PAD symptoms and underwent re-interventions. Newly detected comorbidities and modifications of pharmacotherapy could bias the results. The loss to follow-up and death rate in the eight-year observation was high (combined 35%), and this time point result needs to be interpreted with great caution. Last but not least, the COVID-19 pandemic occurred, which is known to impair the vascular endothelium structure leading to an increase the vascular inflammation. That is why the long-term results should be interpreted with great caution. Second of all, this study was conducted only in patients without unhealing wounds, so the population of chronic limb-threatening ischemia was represented only by patients with Rutherford 4 Stage, so the conclusions should not be implemented in Rutherford 5 and 6 Stage patients.

## 5. Conclusions

The values and the magnitude of changes in the inflammatory biomarkers LTE4 and TXB2 were consistently positively related to life quality during the whole 12-month observation. This leads to the conclusion that the changes in quality of life in patients with peripheral arterial disease are related to the intensity of eicosanoid-based vascular inflammation. The presented results emphasize the role of inflammation in the pathogenesis of atherosclerosis and its influence not only on the blood flow parameters but also directly on the patients-reported outcomes of endovascular treatment. Further studies on the therapies based on the inhibition of vascular inflammation are strongly awaited and desired to improve the existing treatment pathways for patients suffering from peripheral arterial disease.

## Figures and Tables

**Table 1:** Patients’ characteristics.

**Patients Characteristics**		
Participants (*n*, %)	175	100%
Age (mean, SD)	64.77	±8.94
Male (*n*, %)	120	68.57%
Active smoking habit (*n*, %)	47	26.86%
Former smokers (*n*, %)	106	60.57%
**Comorbidities**		
Coronary artery disease (*n*, %)	77	44.25%
Diabetes mellitus t II (*n*, %)	116	66.29%
Hypertension (*n*, %)	128	73.14%
Hypercholesterolemia (*n*, %)	73	41.71%
Heart failure (*n*, %)	21	12.00%
History of MI (*n*, %)	47	26.86%
History of stroke (*n*, %)	17	9.71%
**Peripheral Interventions History**		
No	88	50.29%
Yes, within 1 limb (*n*, %)	49	28.0%
Yes, within both limbs (*n*, %)	38	21.71%
More than 1 restenosis (*n*, %)	32	18.29%

**Table 2:** Spearman correlation of LTE4, TXB2, and 5HETE concentrations and values of VascuQol-6 at each observation timepoint (correlation coefficient; * *p* < 0.01; ** *p* < 0.001).

		VascuQol Preoperative	VascuQol 1 M	VascuQol 3 M	VascuQol 6 M	VascuQol 12 M	VascuQol 8 Y
LTE4	Preoperative	−0.46 **	−0.25 **	−0.26 **	−0.23 **	−0.22 **	0.16
	1 M FU	−0.32 **	−0.55 **	−0.44 **	−0.23 **	−0.24 **	0.08
	3 M FU	−0.35 **	−0.37 **	−0.72 **	−0.41 **	−0.26 **	0.06
	6 M FU	−0.30 **	−0.24 **	−0.42 **	−0.57 **	−0.31 **	0.11
	12 M FU	−0.28 **	−0.18 *	−0.33 **	−0.37 **	−0.71 **	0.03
TXB2	Preoperative	−0.52 **	−0.15 *	−0.24 **	−0.24 **	−0.20 **	0.10
	1 M FU	−0.24 **	−0.21 **	−0.32 **	−0.14	−0.08	−0.01
	3 M FU	−0.27 **	−0.21 **	−0.34 **	−0.21 **	−0.19 *	−0.09
	6 M FU	−0.28 **	−0.21 **	−0.37 **	−0.28 **	−0.20 **	0.02
	12 M FU	−0.16 *	−0.19 *	−0.23 **	−0.21 **	−0.21 **	0.00
5HETE	Preoperative	0.11	0.00	0.11	0.03	0.04	−0.01
	1 M FU	0.06	−0.16 *	0.09	0.12	0.03	0.05
	3 M FU	0.08	−0.19 *	0.03	0.04	0.11	−0.02
	6 M FU	0.05	−0.05	0.07	0.02	−0.08	0.07
	12 M FU	0.05	−0.11	0.11	0.05	0.02	−0.03

**Table 3:** Spearman correlation of LTE4, TXB2, and 5HETE concentrations and VascuQol-6 changes at each observation time point compared to preoperative values (correlation coefficient; * *p* < 0.01; ** *p* < 0.001).

		ΔVascuQol 1 M vs. Preop	ΔVascuQol 3 M vs. Preop	ΔVascuQol 6 M vs. Preop	ΔVascuQol 12 M vs. Preop	ΔVascuQol 8 Y vs. Preop
LTE4	Preoperative	0.13	0.02	0.06	0.04	0.35 **
	1 M FU	−0.21 **	−0.25 **	−0.03	−0.06	0.15
	3 M FU	−0.05	−0.52 **	−0.19 *	−0.06	0.16
	6 M FU	0.02	−0.24 **	−0.37 **	−0.13	0.20 *
	12 M FU	0.06	−0.15 *	−0.17 *	−0.52 **	0.11
TXB2	Preoperative	0.25 **	0.07	0.09	0.09	0.35 **
	1 M FU	0.01	−0.17 *	0.02	0.06	0.17
	3 M FU	0.03	−0.18 *	−0.04	−0.03	0.07
	6 M FU	0.04	−0.20 **	−0.09	−0.04	0.19
	12 M FU	−0.03	−0.12	−0.10	−0.11	0.10
5HETE	Preoperative	−0.08	0.04	−0.04	−0.03	−0.10
	1 M FU	−0.18 *	0.05	0.08	0.00	0.02
	3 M FU	−0.21 **	−0.02	−0.01	0.06	−0.10
	6 M FU	−0.07	0.03	−0.01	−0.11	0.04
	12 M FU	−0.13	0.07	0.02	−0.02	−0.07

**Table 4:** Spearman correlation of changes in LTE4, TXB2, and 5HETE concentrations and VascuQol-6 changes at each observation time point compared to preoperative values (correlation coefficient; * *p* < 0.01; ** *p* < 0.001).

		ΔVascuQol 1 M vs. Preop	ΔVascuQol 3 M vs. Preop	ΔVascuQol 6 M vs. Preop	ΔVascuQol 12 M vs. Preop	ΔVascuQol 8 Y vs. Preop
ΔLTE4	1 M vs. Preoperative	−0.29 **	−0.25 **	−0.07	−0.08	−0.11
	3 M vs. Preoperative	−0.14	−0.53 **	−0.23 **	−0.09	−0.09
	6 M vs. Preoperative	−0.08	−0.24 **	−0.37 **	−0.15	−0.08
	12 M vs. Preoperative	−0.05	−0.18 *	−0.22 **	−0.53 **	−0.17
ΔTXB2	1 M vs. Preoperative	−0.21 **	−0.24 **	−0.06	−0.02	−0.12
	3 M vs. Preoperative	−0.18 *	−0.23 **	−0.11	−0.11	−0.21 *
	6 M vs. Preoperative	−0.16 *	−0.27 **	−0.18 *	−0.12	−0.06
	12 M vs. Preoperative	−0.21 **	−0.17 *	−0.17 *	−0.18 *	−0.11
Δ5HETE	1 M vs. Preoperative	−0.10	0.01	0.12	0.03	0.12
	3 M vs. Preoperative	−0.16 *	−0.06	0.03	0.09	−0.01
	6 M vs. Preoperative	0.00	0.00	0.02	−0.08	0.13
	12 M vs. Preoperative	−0.06	0.05	0.06	0.01	0.02

## Data Availability

The data presented in this study are available on request from the corresponding author. The data are not publicly available due to their ongoing analysis at the moment of publication.

## Supplementary Materials

The following supporting information can be downloaded at: https://www.mdpi.com/article/10.3390/jcm12103412/s1, Figure S1: Concentrations of LTE4, TXB2, and 5HETE at each of the observation time points; Figure S2: Dynamics of LTE4, TXB2, and 5HETE concentrations changes in course of observation period; Table S1: 8-year telemedical follow-up chart.
