# Supplementary material for: Vascular Inflammatory Markers as Predictors of Peripheral Arterial Disease Patients’ Quality-of-Life Changes after Endovascular Treatment
Source: J Clin Med. 2023 May 11;12(10):3412. doi: 10.3390/jcm12103412 (PMC10219135; doi:10.3390/jcm12103412)
Supplement: Supplementary file 1 [file jcm-12-03412-s001.zip › Supplementary Figure S1.pdf]

**Rutherford classification**	☐ 0 ☐ 1 ☐ 2 ☐ 3 ☐ 4 ☐ 5 ☐ 6

**Claudication distance**	metrów

**WIfI scale**	**W** ☐ **I** ☐ **fI** ☐

**Wound**	**Ischemia**	**foot Infection**
☐ **0 – No ulcer or gangrene**<br>☐ **1 – Small or superficial ulcer on leg or foot, without gangrene**<br>☐ **2 – Deep ulcer with exposed bone, joint, or tendon ± gangrene limited to digits**<br>☐ **3 – Deep, extensive ulcer involving forefoot and/or midfoot ± calcaneal involvement ± extensive gangrene**	**Toe pressure:**<br>☐ **0 – ≥60mmHg**<br>☐ **1 – 40-59mmHg**<br>☐ **2 – 30-39mmHg**<br>☐ **3 – <30mmHg**	☐ **0 – Uninfected**<br>☐ **1 – Mild local infection, involving only skin and subcutaneous tissue, erythema 0.5 to ≤ 2 cm**<br>☐ **2 – Moderate local infection, with erythema > 2 cm or involving deeper structures**<br>☐ **3 – Severe local infection with signs of SIRS**

**Subjective change in symptoms compared to postoperative condition**	☐ **Improvement**<br>☐ **Worsening**<br>☐ **Lack of significant change**
**Subjective change in symptoms compared to 12-month follow-up meeting**	☐ **Improvement**<br>☐ **Worsening**<br>☐ **Lack of significant change**

**Major adverse cardiovascular event - MACE**	☐ **NO** ☐ **YES** *(fill below)*

**Type of event**		**Date**
**Death** ☐	**Reason:**	
**Myocardial inferction** ☐ *(fill below)*		
**PCI** ☐ **YES** ☐ **NO**	**CABG** ☐ **YES** ☐ **NO**	
**Stroke** ☐		
**TIA** ☐		

Major adverse limb event - MALE	☐ NO ☐ YES *(fill below)*

Type of event		Date
Major limb amputation	☐	
Minor amputation	☐	
Vascular reintervention	☐	

Height	☐	cm	Weight	☐	kg

Cigarettes smoking	☐ Never<br>☐ In the past<br>☐ Currently	Year of giving up ☐	Years of smoking ☐	Packs per day ☐

Was the life quality assessed?	☐ NO ☐ YES
**Questionnaire**	**Was fully filled?**
Vascular Quality of life – VascuQol-6	☐ YES ☐ NO

Other comments	

Supplementary Figure S1. 8-year telemedical follow-up chart.
