# Supplementary material for: Vascular Inflammatory Markers as Predictors of Peripheral Arterial Disease Patients’ Quality-of-Life Changes after Endovascular Treatment
Source: J Clin Med. 2023 May 11;12(10):3412. doi: 10.3390/jcm12103412 (PMC10219135; doi:10.3390/jcm12103412)
Supplement: Supplementary file 1 [file jcm-12-03412-s001.zip › Supplementary Table S1.pdf]

	N	Mean [µg/ml]	SD
LTE4			
Preoperative	175	149,82	91,64
Postoperative	175	260,24	218,24
1 day after	175	103,97	159,52
1M FU	174	98,18	103,32
3M FU	174	116,24	135,74
6M FU	172	97,28	81,18
12M FU	168	101,97	97,26
TBX2			
Preoperative	175	1760,09	2007,76
Postoperative	175	3157,44	3124,89
1 day after	175	1298,63	1641,39
1M FU	174	1357,49	1404,86
3M FU	174	1498,85	1711,47
6M FU	170	1300,45	1900,54
12M FU	166	1281,72	1408,72
5HETE			
Preoperative	175	81,44	157,73
Postoperative	175	421,05	1081,35
1 day after	175	124,96	320,87
1M FU	174	92,91	238,25
3M FU	174	185,34	577,75
6M FU	171	236,64	856,56
12M FU	168	327,01	846,98

Supplementary Table S1. Concentrations of LTE4, TXB2 and 5HETE at each of the observation time points.
