# Supplementary figures and images for: Vascular Inflammatory Markers as Predictors of Peripheral Arterial Disease Patients’ Quality-of-Life Changes after Endovascular Treatment
Source: J Clin Med. 2023 May 11;12(10):3412. doi: 10.3390/jcm12103412 (PMC10219135; doi:10.3390/jcm12103412)

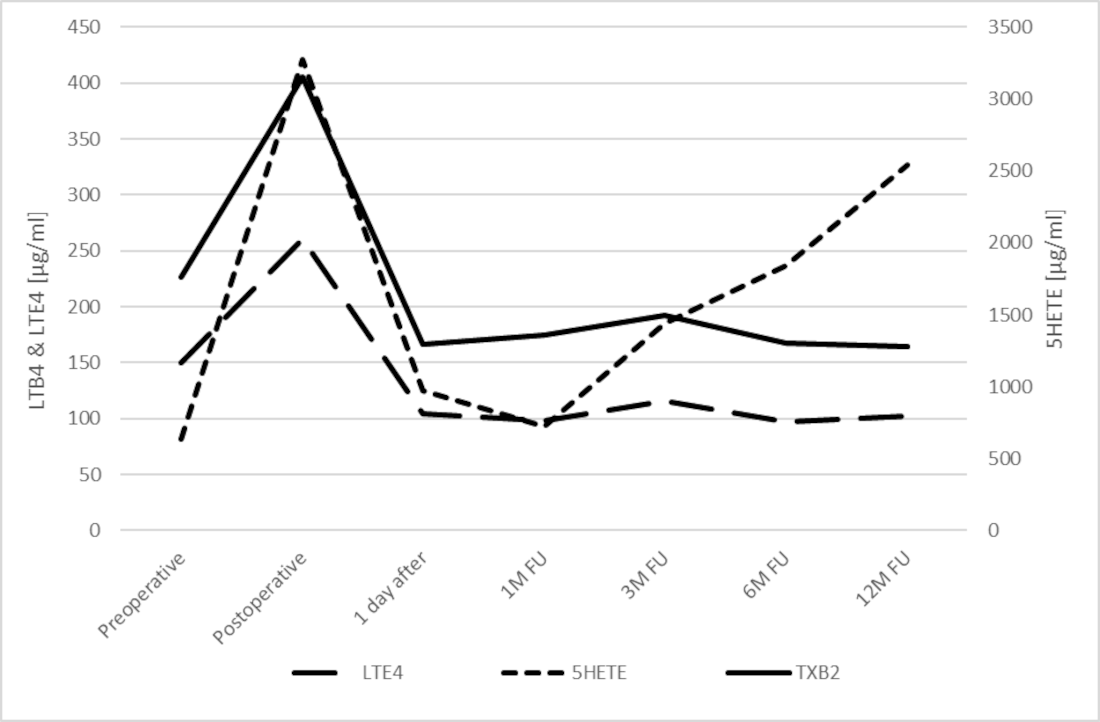

Supplement: Supplementary file 1 [file jcm-12-03412-s001.zip › Supplementary Figure S2.png]
